# Roles of EphA2 Receptor in Angiogenesis Signaling Pathway of Glioblastoma Multiforme

**DOI:** 10.21315/mjms2018.25.6.3

**Published:** 2018-12-28

**Authors:** Wan Noor Ainun Baharuddin, Abdul Aziz Mohamed Yusoff, Jafri Malin Abdullah, Zul Faizuddin Osman, Farizan Ahmad

**Affiliations:** 1 Universiti Teknologi MARA, Shah Alam, 40450 Shah Alam, Selangor, Malaysia; 2 Department of Neurosciences, School of Medical Sciences, Universiti Sains Malaysia, 16150 Kubang Kerian, Kelantan, Malaysia; 3 Centre for Neuroscience Service and Research, Universiti Sains Malaysia, 16150 Kubang Kerian, Kelantan, Malaysia; 4 School of Dental Sciences, Universiti Sains Malaysia, 16150 Kubang Kerian, Kelantan, Malaysia; 5 Human Genome Centre, Universiti Sains Malaysia, 16150 Kubang Kerian, Kelantan, Malaysia

**Keywords:** glioblastoma multiforme, EphA2, angiogenesis

## Abstract

Glioblastoma multiforme (GBM) is one of the most common primary brain tumours in adults, accounting for almost 65% of all cases. Among solid tumours, GBM is characterised by strong angiogenesis, including the highest degree of vascular proliferation and endothelial cell hyperplasia. Despite numerous improvements in existing treatment approaches, the prognosis of GBM patients remains poor, with a mean survival of only 14.6 months. Growing evidence has shown significant overexpression of the ephrin type-A receptor 2 (EphA2) receptor in various malignancies, including GBM, as well as a correlation to poor prognoses. It is believed that EphA2 receptors play important roles in mediating GBM tumourigenesis, including invasion, metastasis, and angiogenesis. Despite the clinical and pathological importance of tumour-associated vasculature, the underlying mechanism involving EphA2 is poorly known. Here, we have summarised the current knowledge in the field regarding EphA2 receptors’ roles in the angiogenesis of GBM.

## Introduction

Ephrin type-A receptor 2 (EphA2) overexpression has been correlated with a poor prognosis in most cancer types, including endometrial (1), colorectal (2), breast (3), ovarian (4), and Glioblastoma multiforme (GBM) (5, 6). Both the development and aggressiveness of these tumours are quite dependent on the modulation of angiogenesis, from which the nutrients needed for tumour cell growth are obtained. The involvement of EphA2 in promoting tumourigenesis has been focused on its roles in cell growth, survival, migration, and invasion (7). Recently, it was also found that EphA2 receptors are significantly involved in modulating tumour angiogenesis (8, 9) and that EphA2 is involved in blood vessel formation and remodeling during the vascular development of cancers (10). In addition, intensive studies from the past few decades have indicated that GBM is one of the most angiogenic solid tumours (11). However, the underlying molecular pathways behind GBM angiogenesis and its aggressiveness remain unclear. Therefore this review discusses how the EphA2 receptor may play a role in GBM angiogenesis.

## Signaling of EphA2 and Ephrin A1 in Tumours

**Figure 1:**
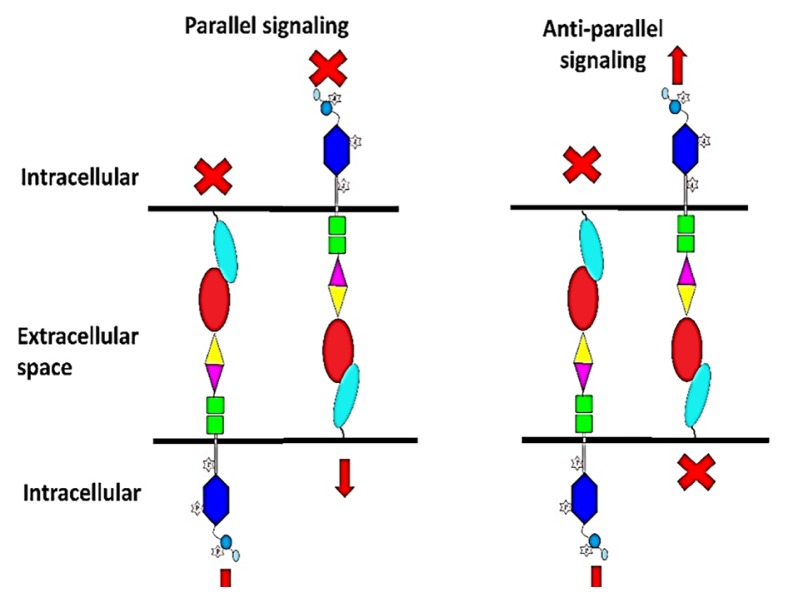
Parallel and anti-parallel signaling of an EphA2-ephrin interaction

The human EphA2 gene is located on chromosome 1, which encodes the 130kDa EphA2 protein with approximately 976 amino acids. It is known that 90% of its sequences are homologous to the EphA2 mouse (12). Despite its ability to interact with various types of ephrins, ranging from types 1 to 5 (13), the most common ligand interaction with the EphA2 receptor is Ephrin A1 (14). Different types of its interaction mode are shown in Figure 1.

Forward signaling is a signal transduction that originates from ephrin ligands and is directed toward Eph receptors. This is also called ephrin:EphA2 forward. Meanwhile, reverse signaling originates from Eph receptors and is directed toward ephrin ligands. This is also called EphA2:ephrin reverse. Due to their membrane localisation, these signals can also be simultaneously activated in both forward and reverse directions, which is known as ephrin-EphA2 bidirectional signaling.

In a situation where two or more Eph-ephrin interactions occur simultaneously, the signaling can be classified as either parallel or anti-parallel. Parallel signaling is induced when forward and reverse signaling occur simultaneously and in parallel to each other, while anti-parallel signaling is a simultaneously occurring forward signaling in which forward ephrin-EphA2 signals are conveyed toward the different directions of the Eph receptor.

The EphA2 receptors could be triggered and activated upon binding to the Ephrin A1 ligand. The membrane attachment of both EphA2 and Ephrin A1 provides various mode mechanisms of interactions that are unique from other receptor tyrosine kinases families. EphA2 and Ephrin A1 can function independently of each other through an interchange with other signaling systems (15). Primarily, interaction Eph receptors with cell-surface tethered-ephrin ligands can activate Eph receptor kinase-dependent signaling. Additionally, the ephrins can also convey signals, which can lead to bidirectional signaling. In this case, the Eph receptor can act as a ligand in the same way that the ligand can act as a receptor (16), and the ephrin cytoplasmic tail enables recruitment of further signaling effectors (17).

The EphA2 receptor phosphorylation and kinase activity have been proven to induce tumour malignancy and confer the cells’ oncogenic potential (18). EphA2 receptors are kinase enzymes that create a signal by transferring a phosphate group to a protein substrate (19). Another mechanism that mediates interactions is the ligand/receptor endocytosis. During endocytosis, Ephrin A1 is cleaved from the cell membrane, and it functionally interacts with EphA2, which leads to signal transduction and receptor activation for the next molecular response (20).

Stimulation of EphA2 causes powerful changes in a tumour cell’s behaviour. Interaction between EphA2 and Ephrin A1, along with other guidance molecules, will navigate developmental guidance that causes sheets of cell layers to become tumours. The contact of Ephrin A1 and the EphA2 receptor in neighboring cells conveys the signal forward, which will cause the cells to repel from each other. If the EphA2 receptor is neither activated nor called as reverse signaling, then the interaction with the ligand will cause either cell adhesion or repulsion (21). As a result, adhesion leads to tissue formation, and repulsion leads to boundary separation between tissues. This same mechanistic action is also being used for tumour formation and invasion.

In addition, ephrin-EphA2 interaction can induce short distance signaling and can mediate cellular processes, such as cell proliferation, migration, formation of tissue boundaries, axon guidance, and platelet aggregation (6, 21, 22). Most importantly, a systematic interaction between EphA2 and ephrin ligands can initiate the formation of a complex network of regulatory pathways that must act coherently to control multifunctioning biological responses.

## Expression of EphA2 in GBM

In brain tumours, high expression of EphA2 is mostly detected in advanced grades of tumours, such as anaplastic astrocytoma and GBM (6). EphA2 is also highly expressed in various types of GBM cell lines, including U87-MG, DBTRG-05M, U251MG, BTCOE 4795, LN229, and T98G (23, 24), as well as in human glioma stem cells (GSC), including D456MG, 827, and 1228 cells. Because EphA2 is known to mediate various key cellular processes, deregulated expressions of its gene and protein in glioma cells enable the promotion of tumour aggressiveness, invasion, and metastasis (25). In the case of GBM, overexpression of EphA2 is linked to low survival rate and tumour recurrence. This appears to hold true when the expression of EphA2 is found in a gradient with a higher expression in the higher grades of gliomas. In a previous study, when compared to benign tumours, higher EphA2 expression was detected in malignant gliomas and significantly correlated to a poorer prognosis (6). It has also been suggested that EphA2 contributes to the malignant transformation of tumours (26).

In addition to being highly expressed in GBM tumour cells, EphA2 expression has also been correlated with the subpopulation of GSC regarding their propagating ability and pool size. It is known that the heterogeneity, molecular genetic make-up, and epigenetics of GBM populations make tumours resilient to current therapeutic strategies. The GSC subpopulation has particularly emerged as one of the key players in GBM recurrence. Specific roles of EphA2 via GSC mediation have shown an interesting relationship with the capacity of the cells to expand their pool size, which ultimately led to GBM recurrence in a previous study. Even though EphA2 receptors’ regulatory functions in GBM tumourigenesis remain limited, knowing that the ability to knock down GSC self-renewal and tumourigenic capabilities exists via modulation of EphA2 expression is intriguing (27).

## Roles of EphA2 in GBM Angiogenesis

Angiogenesis, or the formation of new blood vessels, from the existing vasculature is one of the hallmarks of GBM, which is characterised as a highly vascularised solid tumour. Continuous recruitment of new blood vessels creates a favourable microenvironment in GBM, which allows the malignant transformation of a tumour. Several recent studies have clearly indicated involvement of EphA2 in GBM angiogenesis. Its high expression was found along the tumour vasculature of GBM, suggesting potential roles in neovascularisation (28). While the underlying mechanisms of EphA2 regulation in invasion and metastasis have been elucidated, the knowledge of its participation in the angiogenesis of GBM remains limited.

In general, EphA2 works via Ephrin A1’s signaling axis to regulate multiple events in the transformation of tumour malignancies. This includes the modulation of tumour-associated angiogenesis, which is important to the survival and maintenance of tumour growth. In ovarian cancer and mammary tumourigenesis, EphA2 overexpression has been associated with increased microvascular density, indicating that EphA2 has a role in promoting angiogenesis in the tumour microenvironment (17, 29). Moreover, high expression of EphA2 in the endothelial lining of tumour-associated vasculature in other tumour types has also been documented, including breast cancer and Kaposi’s sarcoma (8).

It has been acknowledged that the EphA2-mediated angiogenesis process in cancer occurs via the recruitment of phosphoinositide 3-kinase (PI3K) and the stimulation of downstream molecules of the Vav family of guanine nucleotide exchange factors (GEFs) and Rac1-GTP. Functionally, this process is partly mediated through crosstalk with pro-angiogenic molecules, such as growth factor receptors and adhesion molecules, including integrins and cadherins (30).

In GBM cell lines, recent evidence has shown that EphA2 regulates vascular endothelial growth factor receptor 2 (VEGFR-2) expression at both the gene and protein levels. Nevertheless, inhibition of EphA2 did not show any impairment of VEGF expression in the same study. It is therefore interesting to suggest that EphA2 may regulate vessel sprouting during developmental angiogenesis independently via VEGFR-2 without affecting VEGF in GBM cells (24). VEGFR-2 is known as the earliest differentiation marker for endothelial cells in the process of vascularisation. Inhibition of VEGFR-2 alone inhibited vessel growth by almost 55%, whereas inhibition of EphA2 alone showed 30% inhibition. Interestingly, simultaneous inhibition of both EphA2 and VEGFR-2 resulted in 95% inhibition of micro vessel growth. These available data suggest that VEGFR-2 and EphA2 signaling pathways play non-redundant roles in angiogenesis (31).

From another perspective, it is intriguing to explore the correlation of EphA2 overexpression in GSC in relation to the GBM angiogenesis process. The GSC population can generally maintain an undifferentiated state, which supports their self-renewal and tumourigenicity. Interestingly, it has been demonstrated that GSCs in GBM tumours can transdifferentiate into endothelial-like cells, which leads to the sprouting of new blood vessels known as vasculogenic mimicry (VM). VM is often detected in high-grade gliomas and has been correlated with poor patients’ prognoses (32). Localised expression of EphA2 was found in VM-positive glioma in comparison to VM-negative glioma, which suggests an association of EphA2 with VM formation. In addition to EphA2, vascular endothelial cadherin (VE-cadherin), which is a transmembrance glycoprotein, is also highly expressed in the GSC subpopulation of GBM. Similar to what has been documented in GBM cell lines, mounting evidence has shown the indispensable roles of VEGFR-2 in the VM formation of GBM; notably, these are independent of VEGF. In such cellular actions, VEGFR-2 is shown to act in combination with Flk-1 signaling to deliver the signal for tubule formation (33).

## Conclusion

GBM are typically aggressive, infiltrative, and resistant to conventional therapies, and poor survival rate is often correlated with tumour recurrence. The first lines of defense in a clinical setting include surgery, chemotherapy, and radiotherapy, which are rarely curative for GBM. Because GBM is one of the most vascularised solid tumours, one potential therapeutic strategy for GBM is targeting angiogenesis. Growing evidence has shown that combination therapy using anti-angiogenic drugs and/or radiotherapy and chemotherapy may be beneficial for treating recurrent GBM. Understanding the molecular pathways behind aberrant blood vessel recruitment in GBM provides an exciting set of potential targets for therapeutic intervention. Overexpression of EphA2 receptors provides a hint for further exploration of the underlying mechanisms behind GBM tumour-associated vasculature formation as well as their crosstalk with other molecules in the signaling pathways. The intricacies of recent studies have not only enhanced our understanding of EphA2 involvement in the pathogenesis and various cellular processes of GBM but also provided an avenue for realising the potential of EphA2 as a therapeutic target for treating malignancies. In addition to its high and localised expression in GBM, very low expression of the receptor is also found in a normal brain, making it an ideal molecular target for medical intervention.
